# Assessment of the Anxiety Level of Andalusian Podiatrists during the COVID-19 Pandemic: The Increase Phase

**DOI:** 10.3390/healthcare8040432

**Published:** 2020-10-26

**Authors:** Samuel Vilar-Palomo, Manuel Pabón-Carrasco, María Luisa Gonzalez-Elena, Lucia Ramírez-Baena, Isabel Rodríguez-Gallego, Aurora Castro-Méndez

**Affiliations:** 1 Hospital Virgen del Rocío, Unidad de Anestesiología y Reanimación, Servicio Andaluz de Salud, Av. Manuel Siurot, SN., 41013 Seville, Spain; samuelvilarpalomo@hotmail.com; 2 Faculty of Nursing, Physiotherapy and Podology, Universidad de Sevilla, C/Avenzoar 6, 41009 Seville, Spain; maruchi1@us.es (M.L.G.-E.); auroracastro@us.es (A.C.-M.); 3 Spanish Red Cross Nursing School, Universidad de Sevilla, Sevilla, Spain, Avda de la Cruz Roja, n° 1 Dpdo, 41009 Sevilla, Spain; luciarb@correo.ugr.es (L.R.-B.); isroga@cruzroja.es (I.R.-G.)

**Keywords:** COVID-19, SARS virus, pandemic, anxiety, psychological

## Abstract

Coronavirus disease (COVID-19) is caused by SARS-CoV-2 and represents the causative agent of a potentially fatal disease that is of great global public health concern. COVID-19 is a respiratory disease produced by the coronavirus family. The World Health Organization declared the disease a pandemic on 11 March 2020. Podiatrists are in a peculiar situation regarding the COVID-19 pandemic: that of a health professional aspect and the singularity that most of them practise as self-employed workers. The aim of the study is to evaluate in a group of podiatrists, working at a national level, their knowledge, perception and degree of anxiety related to the COVID-19 pandemic via the use of a questionnaire specifically developed to this end in the initial phase of the pandemic. We employed a transversal descriptive study with 302 participants, with a purposive sampling technique. The degree of perception and knowledge of the podiatrists about COVID-19 was analysed as well as the cognitive impact of the situation of confinement. The results showed that the podiatrists perceive this situation as serious at the economic and health level, that they have a thorough knowledge of the disease and that they are in a moderate to severe percentile of anxiety. Additionally, 76.2% cancelled their usual work. The COVID-19 pandemic is negatively perceived by this group of podiatrists at the personal, professional, health and economic level, with even a state of anxiety being produced.

## 1. Introduction

On 11 March 2020, the World Health Organization (WHO) declared as a pandemic a respiratory disease due to coronavirus, called COVID-19 [1]. The first cases of the disease were reported on December 2019 in Wuhan (China) and patients were affected by a pneumonia of unknown origin. It was established later that it was an infection caused by a type of virus belonging to the coronavirus family [2]. SARS-CoV-2 is an RNA virus, with a typical crown-like appearance under an electron microscope due to the presence of glycoprotein spikes on its envelope [3]. It is known that are four genera of coronaviruses (CoVs): (I) α-coronavirus (alphaCoV), (II) β-coronavirus (betaCoV), (III) δ-coronavirus (deltaCoV) and (IV) γ-coronavirus (gammaCoV). Previous outbreaks of CoVs include the severe acute respiratory syndrome (SARS) CoV and the Middle East respiratory syndrome (MERS) CoV [3,4,5]. The coronavirus has a zoonotic origin, and diverse studies indicate bats as the main transmitter of the virus to humans [6,7]. However, transmission among humans takes place through contact with people who are carriers of the virus, who act as spreading vectors of it to healthy people [8]. The spread among humans seems to be done through direct contact via respiratory secretions in the form of droplets when coughing, sneezing or speaking, or through contact with these secretions deposited on surfaces and the virus is fundamentally introduced into the organism through the mouth, nose or eyes [9,10,11].

The symptoms of COVID-19 infection appear after an incubation period of approximately 5.2 days [9]. The period from the onset of COVID-19 symptoms to death have ranged from 6 to 41 days, with a median of 14 days [12]. The symptoms shown due to the infection by COVID-19 are varied, from asymptomatic patients who are detected as positive via carrying out a test, to patients who experience a process of bilateral pneumonia which can even jeopardise their vital state, or more common symptoms such as cough, fever and dyspnea [9,13,14,15,16]. In spite of the pathological mechanisms of the disease still being completely unknown, other symptoms which have been revealed are headaches, throat pains, gastrointestinal disorders and vomiting, among others [2,13,15,17]. As to the distribution of the disease according to age and sex, it is estimated that most of the cases (approximately 80%) are in the range of 30 to 69 years of age, the average age being around 58 years old, and 51% of those affected are men [2,9,15,16,18].

The COVID-19 disease can affect certain at-risk groups, such as people over 65 years old with chronic respiratory illnesses, diabetes, heart disease, cancer, and severe obesity or who are immunosuppressed, among others [19,20]. At present, there are no specific antiviral drugs or a vaccine against COVID-19 infection for the potential therapy of humans. The only option available is using broad-spectrum antiviral drugs, such as nucleoside analogues and also HIV protease inhibitors. [21]. Another report showed that the broad-spectrum antiviral remdesivir and chloroquine are highly effective in the control of COVID-19 infection in vitro [22].

In epidemiological terms, according to the WHO, on 7 October, there were 37,594,218 cases confirmed worldwide, distributed over 213 countries, having caused 1,077,326 deaths [23]. In Spain, according to the data of the Ministry of Health, on 7 October 2020, there were 888,968 confirmed cases of COVID-19, of whom 32,929 died [24].

At the national level, since the Spanish government decreed the state of alarm on 14 March 2020 [25], work activity gradually decreased to the point of only allowing that of firms which the government considered essential to maintain basic services during the health crisis. This was regulated through the Royal Decree-Law 10/2020 of 29 March [26].

COVID-19 has had a great impact on the mental health of healthcare professionals. A systematic review published in July 2020, which includes 13 articles analysing the impact of the pandemic on the mental health of nurses, doctors and other health professionals, shows a medium–high level of anxiety in said professionals, in addition to other pathologies such as depression, nervousness and insomnia [27].

In the area of health professions which practise their professional activity privately, this decree-law has had a very significant impact on podiatrists. 

Professional podiatrists have peculiar characteristics with respect to other health professions. Firstly, their main aim is to carry out activities related to the prevention and treatment of foot problems, improving people’s health. Professionals podiatrists are fundamentally self-employed, not generally being included in the public health system, so the current situation has a negative personal and economic impact on them. 

The non-explicit closure of health establishments, as well as the few patients who attended consultations due to the obligatory confinement, has had a negative impact on their incomes. There is also the current obligation to continue with the expenses of maintaining private consultancies (rent, supplies, social security quota, etc.). At the same time, there is the emotional impact of the global pandemic itself. Furthermore, podiatrists do not have the preventative means that health professions at a hospital level have.

Due to the situation of professional podiatrists mentioned above, and in view of the current health crisis because of the COVID-19 pandemic, it is necessary to carry out this study to assess the effects of this situation on the personal and economic level of this group. Based on the results obtained, activities can be carried out to improve the degree of knowledge and decrease anxiety concerning COVID-19 in this health group.

The aim of the study is to evaluate in a group of podiatrists, working in Spain at an autonomic level, the level of anxiety and the relationship that this has with the measures implemented, their work situation and their perception of the pandemic. 

The second objective of the study is to know the degree of knowledge about COVID-19 of this group.

This is done via the use of a questionnaire specifically developed for this purpose in the initial phase of this pandemic. 

H_o_: the anxiety suffered by podiatrists is not related to their perception of their work or personal situation.

## 2. Materials and Method

### 2.1. Research Design

An observational, transversal study with a purposive sampling technique was carried out. The initial chronological phase was from 11 March to 28 March 2020. This was set up with the indications proposed by Strengthening the Reporting of Observational Studies in Epidemiology (STROBE).

### 2.2. Sample

The study population (*n* = 302) was made up of working podiatrists who, after being informed of the nature of the research, voluntarily chose to be part of it. A greater female participation (66.6%) and those licenced in podiatry (65.6%) predominated. The average age was 38.02 years (SD 8.5; range 22–63). Ninety-five percent of the podiatrists were self-employed.

In all the cases, the principles established in the Helsinki Declaration and the agreements with these principles were met [28]. The ethical committee of the Official College of Podiatrists of Andalusia (COPOAN) and Hospital Virgen de Valme, Seville (Spain) CEI approved the study. The date of approval was 24th March of 2020 (1114-N-20).

### 2.3. Procedure and Sampling Technique

The study population, working podiatrists (*n* = 302), was recruited via disseminating the questionnaire through telematic platforms and was coordinated through the Professional Organisation of Podiatrists of Andalusia. The inclusion criteria were: graduates in podiatry currently working in Spain with a self-employed professional activity. 

The information provided by the questionnaire was dealt with anonymously and confidentially with use limited to attaining the aims of this research project. The questionnaire was administered in relation to the progressive rise in the number of those affected by the COVID-19 pandemic. The chronology chosen to carry out this questionnaire coincided with the data published by the Spanish Ministry of Health, Consumption and Social Welfare, where the period of infection with a rising number of cases was indicated, without the highest peak in infections because of the COVID-19 epidemic being reached during the survey [20]. 

Chronology of the data collection: from the 11th to the 28th of March 2020. This was an initial period of infection, the maximum number of infections in Spain had not yet been attained. 

### 2.4. Measures

The sociodemographic variables were compiled via an ad hoc questionnaire concerning age, sex, marital status, level of studies (licenced, graduate, doctor) and the current employment situation (self-employed or employee). The number of minors in the home was considered.

A previous questionnaire used to assess the perceived risk, anxiety and behavioural responses of the general public during the first phase of the influenza A (H1N1) pandemic in the Netherlands was adapted [29]. This questionnaire was previously based an online questionnaire that was based on an existing questionnaire used in studies on risk perception and precautionary behaviours of the general public during outbreaks of SARS [30] and avian influenza [31]. The Cronbach’s alphas of the constructs ranged from 0.7 to 0.9.

A re-adaptation to the new agent was performed and the same structure was maintained. On the other hand, new questions related to the idiosyncrasy of the group were implemented.

In addition, the Anxiety Situations and Responses Inventory (ASRI) was used [32]. The ASRI enables evaluating the cognitive responses of anxiety and detects, in the first instance, physiological and motor responses in specific situations. Its Cronbach’s alpha coefficient is α = 0.90 and it has 12 questions which assess the general level of anxiety (a common feature of an anxious personality). It identifies three systems of independent response (what we think, regulated by the cognitive system; what we feel at the physical level, or physiological system; and what we do, or behavioural–motor system). The minimal obtainable score out of the twelve anxiety answers is, by adding up the scores, zero (0 by 12 symptoms); the maximum score is 48 (4 by 12). 

Finally, the questionnaire had the following structure:

1. Sociodemographic variables: age, sex, nationality.
2. Academic education: licenced, graduate or doctor in podiatry.
3. Professional activity social security system: self-employed or employee.
4. Marital status: single, married, divorced, domestic partnership, widow/er.
5. Block corresponding to knowledge of COVID-19.
6. ASRI questionnaire of anxiety about COVID-19 from the Spanish Society for the Evaluation of Anxiety and Stress.
7. Block corresponding to perceived severity of COVID-19.
8. Block corresponding to perceived effectiveness of the measures before the state of alarm.
9. Block corresponding to perceived effectiveness of the measures after the state of alarm.
10. I consider that…
11. Block corresponding to the information received.
12. Measures taken to avoid infection.

Exploratory analysis of the questionnaire was performed. The Kaiser–Meyer–Olkin (KMO) test and Bartlett’s sphericity test evaluated the applicability of the factor analysis. The statistics show excellent sample adequacy (KMO: 0.905; Bartlett: 1610.29, p: 0.0001), so exploratory factorial analysis of the ASRI questionnaire of anxiety about COVID-19 was carried out. Three components with self-values of more than one were found that explained 67.45% of the variance. For the rotated factorial matrix, the orthogonal rotation method called varimax with Kaiser normalisation (converged on three itineraries) was used. In sums of rotations of squared loads, factor 1 explained 50.51% of variance factor 2, 9.59%, and factor 3, 7.34%. The Cronbach’s alpha coefficient that was obtained was α = 0.85 after translation and re-adaptation.

### 2.5. Statistical Analysis 

The size of the sample was calculated for a power of 0.95, with an alpha error of 0.05 and a size effect of 0.25 (G * Power 3.1.9.4, Franz Faul, University of Kiel, Kiel, Germany). This calculation produced a necessary sample size of 280 subjects. However, the EPIDAT programme was used (expected ratio of 50% and precision of 5%) after finding the number of collegiate podiatrists in 2019 in the community of Andalusia (1500). A sample size of 305 subjects was recommended. Three-hundred and two subjects were finally recruited [33].

Data exploration was done, generating summary statistics for all the cases. This procedure is used to identify atypical or extreme values and characterise differences between groups of cases. Likewise, it enables identifying if the statistical techniques considered are appropriate and indicates the need to transform the data or use non-parametric tests. The numerical variables (quantitative) are summarised with means and standard deviations or, in the case of very asymmetric distributions, through medians and percentiles (P25 and P75), and frequencies and percentages are used for the non-numerical variables (qualitative). The data were analysed using SPSS 24.0 computer software (SPSS Science, Chicago, IL, USA). The Kolmogorov–Smirnov test was applied to determine the distribution of the variables.

The Student’s *t*-test was used to verify the score obtained via the ASRI questionnaire and the parameters. On the other hand, the ANOVA test was used when the qualitative variable was polytomous. Non-parametric tests were used in the case of not meeting normality criteria. The magnitude of differences in pairwise comparisons was tested using the standardised effect size of Cohen’s *d* and eta squared (η^2^). The level of significance adopted for all the statistical analyses was *p* < 0.05 [34].

## 3. Results

### 3.1. Characteristics of the Study Sample

A global sample of 302 subjects was used. An analysis of the sociodemographic and general data of the sample is shown in Table 1.

A descriptive analysis of the competences assessed in the questionnaire was done: “knowledge of COVID-19”, “perceived severity”, “perceived effectiveness of the measures before the state of alarm”, “perceived effectiveness of the measures after the state of alarm”, “information received from the competent authorities” and “perception of the group” (Table 2).

To end the section of descriptive statistics an analysis of the level of anxiety was carried out (Table 3). 

On the other hand, the weighted average of the ASRI questionnaire is 17.66 (SD 10.12, CI 95%, range 16.51–18.80), this indicates that the group has an anxiety level that is between moderate and severe. This is disaggregated into three dimensions, with a weighted average of 8.7 ± 3.5 in the cognitive area, 4.45 ± 4.62 in the physiological area and 4.49 ± 3.09 in the motor dimension. 

### 3.2. Inferential Analysis

An analysis of the level of anxiety and its dimensions with the rest of the parameters studied was done (Table 4). Those variables which have a direct relation with anxiety are related. These were selected in accordance with the descriptive statistics or those sociodemographic variables which could outline the profile of the group. 

The data are statistically significant for the area of work, perception, susceptibility, gender, marital status, treatment received and information given by the government.

## 4. Discussion

As health personnel, professional podiatrists are constantly updated with health knowledge and this is why the results of our study show a high level of knowledge about the main characteristics of the COVID-19 disease. Furthermore, these results are similar to those of other studies where the degree of knowledge of COVID-19 has been analysed in health professionals, such as doctors, nurses and pharmacists [35,36]. This knowledge takes on more importance in the current situation in which it has been revealed that COVID-19 can appear cutaneously in foot injuries. Specifically, these injuries are similar to acro-ischaemic processes associated with the presence of cyanosis and/or tissue necrosis which can be spontaneously resolved [37,38]. Due to this situation, the General Council of Official Colleges of Podiatry in Spain has set up a web page to register compatible cases which can establish the occurrence of this type of injury in patients with a positive COVID-19 diagnosis [39].

The results obtained through our study reveal the impact, at the work level in professional podiatrists, of the measures taken by the Spanish government during the COVID-19 pandemic. A high percentage of them (76.2%) completely cancelled their professional activity, and the rest only deal with urgent consultations. This is why a broad percentage of the study’s participants consider that this situation will have a strong economic impact on their working future. Additionally, due to the decree of the Spanish government not forcing a total closure of podiatry clinics, they are not able to benefit from certain socio-economic measures aimed at palliating the complete closure of firms. 

As to the effectiveness of the measures established in the state of alarm, they are verified as being much more significant once the decree of the state of the alarm was passed. This is due to these measures currently demonstrating their effectiveness in the reduction of infection, hospital/ICU admissions and deaths in comparison with the beginning of the pandemic [24].

The impact of the COVID-19 pandemic on the psychological aspect of health professionals is evident, as has been demonstrated by some studies in which 40% of the health professionals of Wuhan (China) had problems of anxiety, specifically those exposed on the disease’s frontline [40,41]. In turn, another study, carried out in Singapore in February and March, concluded that “non-medical professionals” (health professionals who are neither doctors nor nurses) had a greater level of anxiety than “medical professionals”. This was associated with a greater lack of first-hand information, sufficient protection and training and less access to resources of psychological support to confront the problem [42]. Likewise, other studies of similar characteristics in diseases such as the H1N1 virus and severe acute respiratory syndrome (SARS) established high levels of anxiety in health professionals on the disease’s frontline, as well as a directly proportional relation between the information received and the perceived anxiety [43,44].

There were significant differences regarding gender. Greater anxiety is shown in women than in men. These results coincide with the study conducted by Lai et al. In this study, we observed greater anxiety in female professionals [41]. However, the healthcare professionals are predominantly female, therefore, this may be a bias to take into account. 

Regarding age, in our study, there were no significant differences. On the other hand, Huang et al. concluded that younger people reported a significantly higher prevalence of generalised anxiety disorder and depressive symptoms than older people [45]. Our results may be related to the false feelings of protection on the part of young people against COVID-19. The latest data show a decrease in the average age of those infected.

Finally, marital status was a variable that influenced this population. People who live with other family members have a higher perception of risk. There is no doubt that healthcare professionals have needs at the family level for health, knowledge and safety that are increased in pandemic situations, and may affect their professional performance. The personal concern most frequently related to the COVID-19 outbreak is that related to family health, the risk of transmitting the disease to their family and their patients [27,46].

The data contributed by these studies can be related with the results obtained in our study, which establish a significant level of anxiety in professional podiatrists concerning the current situation. Specifically, this anxiety is fundamentally marked by marital status, with the majority of the respondents being married. This could be due to the worry about having the disease and infecting their partner and family, as well as the lack of an economic income essential for the whole family. The perceived susceptibility and the possibility of getting infected are situations which also breed a huge sense of powerlessness in professional podiatrists. This increases their levels of anxiety as well.

This research reveals the shortages suffered by health staff who are not backed by the public health system. Additionally, there is the aggravating fact that their professional practice takes place totally in the private sector. Emergencies do not overcome the economic needs of these professionals. Added to this is that a high percentage of their users are people at risk (the elderly) and that these people must rigorously maintain social distancing. Therefore, this group should be included in the economic help given to the self-employed, thus avoiding social and economic deprivation. On the other hand, these results can be extrapolated to professions whose characteristics are similar (physiotherapists, dentists, etc.). One must not forget that these groups give an important service to the population and a region’s care quality depends on their survival. 

A limitation of our study is not having a control group for comparison with a non-health group or a comparison with health workers practising in the public sector. 

## 5. Conclusions

Podiatry professionals show a broad knowledge of the clinical characteristics of COVID-19 in relation to updated information on the disease and other studies on the knowledge of health professionals, and are also health personnel able to detect foot symptoms associated with it. The measures adopted by the government regarding their work situation is not to their liking. 

Likewise, they are affected by a high level of anxiety (the situation breeds a sense of powerlessness) associated with the COVID-19 pandemic due to its economic impact as they are self-employed. As well as not being able to generate necessary incomes, they have the possibility of suffering from or transmitting the disease in their environment. These data can be extrapolated to professionals with identical work situations, such as dentists and physiotherapists.

## Figures and Tables

**Table 1:** Sociodemographic and general data of the sample.

Category (*n* = 302)	Subcategories	% (*n*)
Gender	Men	33.44 (*n* = 101)
	Women	66.55 (*n* = 201)
University studies	Licenced in podiatry	64.5(*n* = 195)
	Graduated in podiatry	30.5 (*n* = 92)
	Doctor (PhD)	5.0 (*n* = 15)
Employment situation	Self-employed	78.1 (*n* = 236)
	Self-employed with employees	4.6 (*n* = 14)
	Employee	17.2 (*n* = 52)
Marital status	Single	27.5 (*n* = 83)
	Married/Living together	66.2 (*n* = 200)
	Divorced/Widow(er)	6.3 (*n* = 19)
Children < 18 years old in the home	Yes	51.7 (*n* = 156)
	No	48.3 (*n* = 146)
I have completely cancelled my professional activity	Yes	76.2 (*n* = 230)
	No	23.8 (*n* = 72)
I believe this will have an economic impact on my work future	Yes	96.4% (*n* = 291)
	No	2.0% (*n* = 6)
	Perhaps	1.7% (*n* = 5)
In the case of continuing with the professional activity	I am only dealing with emergencies	100% (*n* = 302)
	I continue dealing with my patients as normal	0.0% (*n* = 0)
I consider the information received from the competent health authorities to be sufficient	Yes	7.6% (*n* = 23)
	No	92.4% (*n* = 279)
Due to the measures taken by the government, I feel abandoned as a member of a professional group	Yes	95% (*n* = 287)
	No	1.3% (*n* = 4)
	
	Perhaps	3.6% (*n* = 11)

**Table 2:** Descriptive analysis of the dimensions evaluated.

Knowledge	YES	NO	DK/DA		
1. COVID-19 is caused by a new virus	81.1%	16.6%	2.3%		
2. A vaccine against COVID-19 is available	1.0%	99.0%	0.0%		
3. COVID-19 can be transmitted by human to human contact	96.0%	3.6%	0.3%		
4. The symptoms of COVID-19 are visible in all the cases	2.0%	98.0%	0.0%		
5. Pets transmit COVID-19	1.0%	91.4%	7.6%		
6. COVID-19 is transmitted by wild animals	41.7%	35.8%	22.2%		
7. It has a virulence similar to conventional influenza	47.4%	49.7%	3.0%		
Perceived severity	Very high	High	Medium	Low	Very low
8. Severity of COVID-19	22.8%	44.4%	28.8%	1.7%	2.3%
9. Risk of contracting the disease because of my age or presence of previous pathologies	6.0%	18.2%	21.2%	18.9%	35.8%
10. COVID-19 is harmful for my health	37.1%	22.2%	27.2%	9.3%	4.3%
11. Perceived susceptibility	11.6%	24.2%	39.7%	14.9%	9.6%
12. Perceived possibility of getting infected	22.8%	28.1%	27.5%	17,2%	4.3%
13. Perceived possibility of getting infected in comparison with others	19.5%	27.8%	26.5%	12.9%	13.2%
Perceived effectiveness of the measures before the state of alarm	Very high	High	Medium	Low	Very low
14. Avoid crowded places	67.5%	5.0%	13.9%	8.6%	5.0%
15. Carry out better hygiene	78.8%	11.3%	7.0%	2.3%	0.7%
16. Use of mask	51.0%	14.9%	18.9%	8.9%	6.3%
17. Seek medical attention if flu symptoms appear	37.7%	15.2%	27.2%	9.9%	9.9%
18. Stay at home	74.8%	9.6%	9.6%	2.6%	3.3%
19. Continue with my work activity, increasing the prevention measures	15.6%	9.6%	18.2%	15.9%	40.7%
Perceived effectiveness of the measures after the state of alarm	Very high	High	Medium	Low	Very low
20. Avoid crowded places	89.7%	5.0%	4.0%	1.0%	0.3%
21. Carry out better hygiene	93.7%	5.0%	1.3%	0.0%	0.0%
22. Use of mask	60.9%	17.2%	13.2%	4.6%	3.3%
23. Seek medical attention if flu symptoms appear	48.3%	17.5%	22.5%	6.6%	5.0%
24. Stay at home	80.5%	8.9%	7.9%	1.0%	1.7%
25. Continue with my work activity, increasing the prevention measures	25.2%	7.3%	12.3%	11.6%	43.7%
Perception	Totally agree	Agree	Neither agree nor disagree	Disagree	Totally disagree
26. The threat is exaggerated by the media and the government	8.6%	7.9%	10.6%	12.9%	59.9%
27. The situation is worse than what was predicted	86.1%	8.3%	2.6%	1.0%	2.0%
28. There is nothing we can do about it	10.3%	10.6%	19.5%	14.6%	45.0%
29. The situation breeds a sense of powerlessness	76.5%	10.3%	7.0%	3.6%	2.6%
30. Confinement is the only option	58.3%	22.2%	10.9%	4,6%	4.0%
31. As a health professional I consider the information received by the government to be reliable	0.7%	6.6%	17.5%	27.5%	47.7%

Note: DK/DA: do not know/did not answer.

**Table 3:** Valuation of the level of anxiety.

Perceived Anxiety. Anxiety Situations and Responses Inventory (ASRI)	Almost Always	Often	At Times	Not Often	Almost Never
1. Worried about the COVID-19 epidemic	50.7%	33.8%	12.3%	3.3%	0.0%
2. Negative thoughts or feelings about myself	14.6%	29.1%	34.8%	13.9%	7.6%
3. Afraid that my anxiety will be noted and of what people will think if that happens	11.3%	17.5%	24.8%	14.9%	31.5%
4. Stomach trouble	4.0%	11.3%	20.5%	12.3%	52.0%
5. Sweating	0.7%	6.3%	12.3%	12.3%	68.5%
6. Shaking	0.0%	2.6%	8.3%	11.6%	77.5%
7. Tension	7.9%	15.9%	21.5%	13.9%	40.7%
8. Palpitations, fast heartbeat	6.6%	9.9%	18.2%	16.6%	48.7%
9. Repetitive movements (feet, hands, scratching myself)	4.6%	13.6%	14.9%	11.9%	55.0%
10. Smoking, eating or drinking in excess	36.4%	18.2%	20.2%	12.3%	12.9%
11. Avoiding situations	16.9%	24.8%	22.8%	14.6%	20.9%
12. Insecurity	38.4%	20.2%	18.2%	12.3%	10.9%

**Table 4:** Analysis of the anxiety of the podiatrists.

	Perceived Anxiety (ASRI) Total	SE	Cognitive (ASRI)	Physiological (ASRI)	Motor (ASRI)
	χ 2 o u (*p*)		χ 2 o u (*p*)	χ 2 o u (*p*)	χ 2 o u (*p*)
Gender ^α^	6765 (0.001 ***)	0.06 ^&^	6929 (0.001 ***)	7478 (0.001 ***)	7854 (0.002 ***)
Level of studies ^†^	2.30 (0.316)	0.01 ^#^	1.99 (0.369)	5.76 (0.055)	5.38 (0.068)
Employment situation ^†^	0.512 (0.774)	0.04 ^#^	0.532 (0.776)	4.01 (0.135)	1.39 (0.498)
Marital status ^†^	6.38 (0.041 *)	0.02 ^#^	5.30 (0.070)	3.72 (0.155)	6.62 (0.032 *)
Children < 18 years in the home ^α^	10907 (0.658)	0.01 ^#^	11260 (0.953)	11,145 (0.831)	10644 (0.379)
I have completely cancelled my professional activity ^†^	5.53 (0.019 **)	0.02 ^#^	2.23 (0.135)	8.96 (0.003 ***)	4.26 (0.039 *)
I believe it will have an economic impact on my work future ^†^	8.56 (0.014 *)	0.03 ^#^	5.18 (0.075)	9.36 (0.009 **)	7.23 (0.027 *)
I consider the information received from the competent health authorities to be sufficient ^†^	21.36 (0.001 *)	0.03 ^#^	19.42 (0.001 *)	13.46 (0.001 *)	9.63 (0.002 *)
Severity of COVID-19 ^†^	40.3 (0.001 ***)	0.13 ^&^	52.4 (0.001 ***)	14.9(0.005 **)	24.7 (0.001 ***)
Risk of contracting the disease because of my age or presence of previous pathologies ^†^	38.3 (0.001 ***)	0.12 ^&^	39.6 (0.001 ***)	30.0 (0.001 ***)	25.1 (0.001 ***)
COVID-19 is harmful for my health ^†^	38.2 (0.001 ***)	0.11 ^&^	40.1 (0.001 ***)	24.4 (0.001 ***)	34.1 (0.001 ***)
Perceived susceptibility ^†^	100.3 (0.001 ***)	0.31 ^$^	98.7 (0.001 ***)	68.5 (0.001 ***)	67.5 (0.001 ***)
Perceived possibility of getting infected ^†^	75.3 (0.001 ***)	0.23 ^$^	77.5 (0.001 ***)	54.6 (0.001 ***)	48.6 (0.001 ***)
Perceived possibility of getting infected in comparison with others ^†^	55.1 (0.001 ***)	0.18 ^$^	45.5 (0.001 ***)	43.7 (0.001 ***)	41.5 (0.001 ***)
The threat is exaggerated by the media and the government ^†^	26.1 (0.001 ***)	0.18 ^$^	23.1 (0.001 ***)	14.4 (0.001 ***)	21.4 (0.001 ***)
The situation is worse than what was predicted ^†^	14.2 (0.007 **)	0.04 ^#^	12.4 (0.014 *)	10.46 (0.031 *)	8.4 (0.076)
There is nothing we can do about it ^†^	3.54 (0.472)	0.01 ^#^	8.49 (0.075)	3.38 (0.496)	2.38 (0.655)
The situation breeds a sense of powerlessness ^†^	32.9 (0.001 ***)	0.11 ^&^	20.9 (0.001 ***)	28.2 (0.001 ***)	27.4 (0.001 ***)
Confinement is the only option ^†^	22.8 (0.001 ***)	0.11 ^&^	25.1 (0.001 ***)	15.2 (0.004 **)	18.2 (0.001 ***)
As a health professional I consider the information received from the government to be reliable ^†^	13.3 (0.011 *)	0.11 ^&^	6.7 (0.153)	22.2 (0.001 ***)	13.4 (0.009 **)

Note: ^†^ Kruskal–Wallis test; ^α^ Mann–Whitney U; ES: effect size.; ^&^ small ES; ^#^ medium ES; ^$^ large ES. Significance set at *p* < 0.05. * *p* <0.05; ** *p* <0.01; *** *p* <0.001.
